# Effects of Erector Spinae Plane Block and Quadratus Lumborum Block on Postoperative Opioid Consumption in Laparoscopic Kidney Surgery: A Randomized Controlled Clinical Trial

**DOI:** 10.1155/prm/8869716

**Published:** 2025-08-04

**Authors:** Tomasz Skladzien, Pawel Maciejewski, Micha Cicio, Wojciech Szpunar, Jan Szpor, Tomasz Lonc, Anna Kwinta, Renata Bugielska, Olga Szkudlarek, Tomasz Drygalski, Michal Terlecki

**Affiliations:** Department of Intensive Interdisciplinary Care, Jagiellonian University-Collegium Medicum, Cracow, Poland

**Keywords:** anesthesia, erector spinae plane block, pain measurement, postoperative analgesia, quadratus lumborum block, regional, ultrasound-guided regional anesthesia

## Abstract

**Background:**

The quadratus lumborum block (QLB) and erector spinae plane (ESP) block are relatively new regional analgesic techniques that provide analgesia to the abdominal wall and reduce postoperative opioid consumption. We compared the effectiveness of ultrasound-guided bilateral ESP block versus bilateral QLB in patients undergoing laparoscopic kidney surgery.

**Methods:**

Adult patients who underwent laparoscopic nephrectomy or nephron-sparing surgery (NSS) within the study period were included. Patients were randomly assigned to one of two groups: group I received an ultrasound-guided ESP block with 30 mL of 0.35% ropivacaine on each side and group II received an ultrasound-guided QLB 1 with 30 mL of 0.35% ropivacaine on each side.

**Results:**

A total of 84 patients were included, with 45 patients in the ESP block group and 39 in the QLB group. The mean dosage of oxycodone in the ESP block group was 22.66 mg and in the QLB group was 22.66 mg. There was no difference in oxycodone consumption within the first 24 h after surgery between the groups (*p*=0.77).

**Conclusion:**

The effect of ultrasound-guided bilateral QLB and ESP blocks in patients undergoing laparoscopic kidney surgery was found to be similar in terms of postoperative pain and opioid consumption. There were no significant differences between the blocks in opioid consumption or pain scores. Both techniques appear to be effective and safe components of multimodal analgesia strategy for laparoscopic nephrectomy.

**Trial Registration:**

ClinicalTrials.gov identifier: NCT05446727

## 1. Introduction

Laparoscopic nephrectomy, either partial or radical, is the most common surgical procedure for renal cancer [1]. It has been shown that laparoscopic surgery, when compared to POEM surgery, is associated with a significant reduction in postoperative pain and opioid consumption, lower morbidity, faster recovery, and a shorter hospital stay [2]. Pain is an important aspect of perioperative care, and the use of analgesic treatment is aimed at facilitating early postoperative recovery. Traditionally, an analgesic protocol consisting of a multimodal analgesic approach, including regional analgesia, nonopioid agents, and opioids, provides satisfactory pain control in most patients. Unfortunately, opioid analgesics have several short- and long-term side effects that can complicate and prolong surgical recovery and/or increase healthcare costs (e.g., nausea, vomiting, and ileus) [3].

Moreover, the immunosuppressive effect of opioids could be particularly relevant in oncologic patients, potentially increasing the risk of infection and cancer recurrence [4]. The effective application of an analgesic technique plays an important role in improving patient satisfaction and quality of life after surgery. Regional anesthesia techniques have been used to alleviate pain and optimize enhanced recovery after laparoscopic nephrectomy, aiming to avoid or decrease the need for opioids [5].

The quadratus lumborum block (QLB) was first described by Blanco in 2007 [6] and later subdivided into three basic approaches known as QLB 1, 2, and 3, along with a modified approach called the intramuscular QLB. These approaches are distinguished by the needle tip position and the spread of local anesthetic (LA) [7]. In the QLB1 (lateral) approach, LA is deposited anterolateral to the QLB muscle, primarily targeting the T12-L1 nerve branches (subcostal, iliohypogastric, and ilioinguinal nerves). In contrast, the QLB2 (posterior) approach involves injection more posteriorly, between the QLB and latissimus dorsi, aiming for spread to the thoracolumbar fascia and paravertebral space to cover T7–T12 nerves. Recently, numerous case reports and randomized trials have shown that QLB may be effective in relieving postoperative pain and providing an opioid-sparing effect after various surgical procedures [8, 9].

The erector spinae plane (ESP) block is a novel interfascial plane block used for postoperative and chronic neuropathic pain relief in the thoracoabdominal region. Despite its initial indication for the treatment of chronic pain, it has recently been used as a postoperative regional analgesia technique in various surgeries [10, 11]. The block targets the dorsal and ventral rami of the thoracic and abdominal spinal nerves to provide analgesia for multiple types of surgical procedures and painful conditions. Because the ESP block is easier to perform and located far from vital organs and blood vessels, the risks of nerve damage, pneumothorax, and other complications are lower [12].

Several recent studies have directly compared ESP and QLB in different surgical settings, yielding mixed results. Aygun et al. [13] compared ESP and QLB after laparoscopic cholecystectomy and found no significant difference in opioid consumption or most pain scores between the blocks. Zhang et al. [14] reported that an ESP block reduced 24 h opioid requirements by about 30% compared to a QLB (using the QLB2 approach) in laparoscopic nephrectomy patients. In contrast, Onay et al. [15] observed no analgesic differences between ESP and QLB2 in open nephrectomy, although both provided effective pain relief versus control group. Notably, the type of QLB performed may account for some discrepancies: for instance, Zhang's study used a posterior QLB (QLB2), whereas our study (and Aygun's [13]) used the lateral QLB1 approach. The pain profile of laparoscopic kidney surgery includes somatic pain from port-site incisions and visceral pain from kidney mobilization and resection. Both QLB and ESP blocks have the potential to address these components—the QLB (especially QLB1) by blocking abdominal wall nerves and possibly spreading to the paravertebral space for visceral nerve inhibition and the ESP block by spreading extensively in the paravertebral plane to block both dorsal rami and rami communicantes of the spinal nerves.

Our primary aims were to compare postoperative pain intensity using the Numeric Rating Scale (NRS) and the total amount of opioid consumption in the first 24 h after laparoscopic renal surgery between patients who received the ultrasound-guided bilateral ESP block and those who received bilateral QLB.

## 2. Methods

This was a single-center, prospective randomized controlled trial involving patients who underwent either partial or radical laparoscopic nephrectomy in “[Anonymized]” from May 2022 to December 2023. The Bioethics Committee “[Anonymized].” And all methods adhered to the principles outlined in the 2013 Declaration of Helsinki. The study was prospective and registered at ClinicalTrials.gov (“[Anonymized]”). All study participants provided written informed consent to participate voluntarily.

### 2.1. Inclusion and Exclusion Criteria

Adult female and male patients over 18 years old with a body mass index (BMI) < 45 kg/m^2^, who underwent laparoscopic nephrectomy or nephron-sparing surgery within the study period, were included in the study and classified as below American Society of Anesthesiologists (ASA) risk class IV.

We excluded the following patients:

- Patients who refused to participate in the study.
- Patients with contraindications to the blocks, such as infection at the site of needle insertion, empyema, a tumor occupying the thoracic paravertebral space, coagulopathy, and bleeding disorders, or therapeutic anticoagulation.
- Patients unable to use a patient-controlled analgesia (PCA) pump due to comprehension barriers.
- Patients with known allergies to LAs.
- Patients with chronic pain syndromes.
- Patients with chronic opioid use patients who experienced intraoperative complications requiring conversion to open surgery (such as massive hemorrhage).

The BMI threshold of < 45 was chosen to avoid technical difficulties and potential alterations in block efficacy in morbidly obese patients.

Patients were randomized in a 1:1 ratio to one of two groups (ESP or QLB) prior to surgery. The randomization sequence was generated using Microsoft Excel 2016 with a random number function. Allocation concealment was ensured using sequentially numbered, opaque-sealed envelopes prepared by a researcher not involved in patient care. These envelopes were opened only after patient enrollment on the day of surgery, just before block administration. The anesthesiologist performing the block was necessarily unblinded to the group assignment due to the nature of the intervention. However, the patients were not informed of which block technique was used, and all surgeons, recovery room staff, and outcome assessors were blinded to group allocation.

Patients were randomized before laparoscopic kidney surgery and assigned to one of two groups: Group I received an ultrasound-guided ESP block with 30 mL of 0.35% ropivacaine on each side, while group II received an ultrasound-guided QLB1 with 30 mL of 0.35% ropivacaine on each side. Both groups received their blocks before undergoing laparoscopic surgery.

We chose a ropivacaine concentration of 0.35% to balance volume and dose—this was achieved by diluting 0.5% ropivacaine with normal saline. Each 30 mL injection contained 105 mg of ropivacaine (210 mg total for bilateral blocks), which is within accepted safe limits for the adult patients in our study. No signs of LA systemic toxicity were observed.

### 2.2. ESP Block Technique

**Figure 1:**
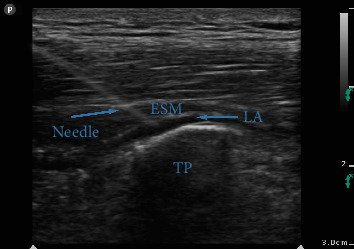
ESP block as seen on ultrasound examination. The transverse process (TP) is visible as a hyperechoic protuberance with acoustic shadowing, and the erector spinae muscle (ESM) lies above it. Blue arrows indicate the block needle advancing in-plane toward the TP, and the spread of local anesthetic (LA) can be seen in the plane deep to the ESM.

Patients were placed in the sitting position for the ESP block. After skin disinfection, a linear high-frequency (L14-6NS) ultrasound probe (Mindray, UMT-400, Mindray Building, Keji 12^th^ Road South, High-Tech Industrial Park, Nanshan, Shenzhen, People's Republic of China), covered with a sterile sheath, was positioned sagittally 1–2 cm lateral to the midline at the level of the ninth thoracic vertebra (T9). The T9 level was earlier identified by palpating the spinous process of the seventh cervical vertebra and counting down nine intervertebral spaces. After identifying the erector spinae muscle (ESM) and transverse process (TP), a 21-gauge needle (Echoplex + REF 6194.853, 85 mm) was inserted deep into the ESM in a craniocaudal direction, using an in-plane technique (Figure 1). The needle was advanced, ensuring it crossed all muscle layers until it contacted the TP. Correct needle placement was confirmed by administering 0.5–1 mL of LA. After ensuring negative aspirations for blood, a total of 30 mL of 0.35% ropivacaine was slowly injected deep to the ESM. This procedure was performed bilaterally on each patient.

### 2.3. QLB Technique

**Figure 2:**
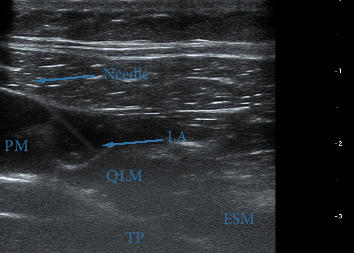
QLB as seen on ultrasound examination. The quadratus lumborum muscle (QLM) is visualized adjacent to the transverse process (TP), with the psoas major (PM) and erector spinae muscle (ESM) visible in deeper and superficial planes, respectively. Blue arrows indicate the block needle advancing in-plane from lateral to medial, targeting the fascial plane anterior to the QLM. The spread of local anesthetic (LA) is visualized as a hypoechoic layer between the QLM and the transversalis fascia.

Patients were placed in the lateral decubitus position. After skin disinfection, a linear high-frequency (L14-6NS) ultrasound probe (Mindray, UMT-400, Mindray Building, Keji 12^th^ Road South, High-Tech Industrial Park, Nanshan, Shenzhen, People's Republic of China), covered with a sterile sheath, was positioned above the iliac crest and moved cranially until the three abdominal wall muscles were clearly identified. The probe was then slid medially until latissimus dorsi and QLM muscles were visualized within identical short-axis views. A 21-gauge needle (Echoplex + REF 6194.853, 85 mm) was inserted from the edge of the probe using an in-plane technique and advanced into the fascia over the QLM. After ensuring negative aspirations for blood, 30 mL of 0.35% ropivacaine was then injected slowly into the fascial interspace between the QLM and internal oblique muscles (Figure 2). This procedure was performed bilaterally on each patient by an experienced anesthesiologist.

### 2.4. Definition of Studied Groups

The ESP group consisted of patients who had bilateral ESP block, while the QLB group consisted of patients who received bilateral QLB.

### 2.5. Induction and Maintenance of Anesthesia

Standard monitoring during the procedure included arterial oxygen saturation, ECG, and noninvasive arterial blood pressure. Premedication was not used. Standard anesthesia consisted of propofol (1.5–2 mg/kg body weight IV) for induction and an infusion of remifentanil using a plasma target-controlled infusion (TCI) with a calculated plasma level of 1–6 ng/mL. Rocuronium (0.6 mg/kg body weight IV) was administered prior to intubation. Neuromuscular blockade was ensured throughout the surgical procedure using rocuronium, guided by the TOF index for adequate muscle relaxation. Anesthesia was maintained with desflurane (MAC 1–1.5). In all groups, desflurane end-tidal concentrations were titrated according to the instantaneously registered electroencephalography monitor to achieve the Bispectral Index (BIS) value between 40 and 60. Mechanical ventilation was delivered with a tidal volume of 6–8 mL/kg of ideal body weight. The respiratory rate was adjusted to maintain the end-tidal carbon dioxide (etCO_2_) concentration within the 35–45 mmHg range. PONV prophylaxis was achieved with intravenous dexamethasone (0.1 mg/kg IV) at induction and intravenous ondansetron (0.1 mg/kg IV) 30 min before the end of surgery.

### 2.6. Surgical Procedure

All surgeries were performed laparoscopically via a transperitoneal approach. Typically, four trocars were used. The first port (camera port) was placed on the side of the operated kidney, above the umbilicus, through a small incision in the rectus abdominis (mini-laparotomy). Three additional ports were inserted as follows: one in the epigastric region (midline, below the xiphoid), and two along the midclavicular line (one at the level of the umbilicus or mesogastrium, and one more cephalad in the subcostal area if needed). The kidney specimen (for radical nephrectomy or the resected tumor for partial nephrectomy) was extracted through the enlarged incision used for the first port. All patients received similar surgical techniques by experienced urologists to minimize variability in pain due to surgical factors.

### 2.7. Postoperative Analgesia

Before surgery, all patients received a combination of intravenous analgesics: magnesium sulfate 2 g, paracetamol 1 g, and metamizole 2.5 g. This regimen was repeated every 6 h for paracetamol and every 12 h for metamizole. Additionally, all patients received 2 mg of intravenous oxycodone 15 min before the end of surgery. Prior to surgery, all patients were given instructions on the functions of the PCA pump (B Braun Perfusor Space, REF 8713030, SN 444655, B Braun Melsungen AG, 34209 Melsungen, Germany). The intravenous PCA pump was programmed to deliver a standard bolus of 2 mg of oxycodone only on demand with a 10-min lockout time. Patients were instructed to use the pump if the NRS (0–10/10) score was ≥ 4.

### 2.8. Primary Endpoint

As the primary outcome parameter, we evaluated the cumulative oxycodone consumption within the first 24 postoperative hours.

### 2.9. Secondary Endpoints

Secondary outcome parameters included (1) pain intensity at 1, 2, 6, 12, and 24 h after surgery, assessed using the NRS (0–10, where 0 = no pain and 10 = worst pain imaginable) at rest; (2) the incidence of PONV during the first 24 h post-op (defined as any report of nausea or any episode of emesis, treated with rescue antiemetics as needed); (3) the total dose of remifentanil administered intraoperatively; (4) estimated intraoperative blood loss (EBL, in mL); (5) the total volume of IV fluids given intraoperatively; and (6) time to awake, defined as the time from surgery end to eye opening on command (or extubation). We also observed and recorded any block-related complications (e.g., LA toxicity signs, hematoma, intravascular injection) and any other adverse events such as excessive sedation, respiratory depression, or allergic reactions.

### 2.10. Data Analysis

A power analysis was performed based on preliminary data. A pilot set of 10 patients (5 per group) suggested a mean 24 h oxycodone consumption of 17 mg (SD ± 13) in the ESP group and 25 mg (SD ± 7) in the QLB group. Using these figures, we calculated that a minimum of 54 patients (27 per group) would be required to detect a statistically significant difference with 80% power at the *α* = 0.05 level (two-tailed). To account for potential dropouts or exclusions, we aimed to enroll approximately 90 patients. The sample size was increased to 87 patients, of whom 84 completed the study.

The analysis of quantitative variables (e.g., those expressed in numbers) was performed by calculating descriptive statistics such as mean, standard deviations, median, quartiles, minimum, and maximum values. The analysis of qualitative variables (e.g., those not expressed in numbers) was performed by enumerating absolute frequencies and percentages of occurrences for all possible values of these variables. The comparison of qualitative variable values between two groups was performed using the chi-square test (with Yates's correction for 2 × 2 tables) or Fisher's exact test when the assumptions for the chi-square test regarding the expected frequencies were not met. The comparison of the quantitative variable values between two groups was performed using the Mann–Whitney *U* test. Correlations between quantitative variables were analyzed using Spearman's rank correlation coefficient. A one-way analysis of the influence of quantitative characteristics on a dichotomous variable (e.g., taking only two possible values—occurrence or absence of extensive bleeding) was performed using logistic regression. The significance level of 0.05 was adopted for the analysis, so all *p*-values below 0.05 were interpreted as indicating significant relationships. To account for the risk of type I error due to multiple comparisons between baseline variables, a Bonferroni correction was applied. Since four independent demographic and anesthetic characteristics were compared between the study groups, the threshold for statistical significance was adjusted to *α* = 0.0125 (0.05/4). Therefore, *p*-values below 0.0125 were interpreted as statistically significant for intergroup differences in baseline characteristics. The analysis was performed in R, version 4.3.2 [16].

## 3. Results

**Figure 3:**
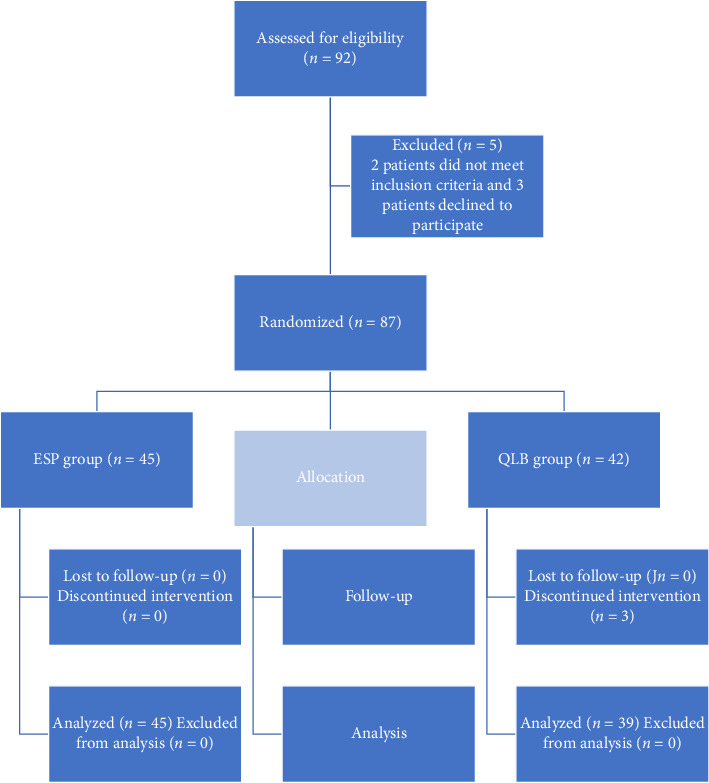
Consort flowchart of the study.

From May 2022 to December 2023, 92 participants underwent laparoscopic kidney surgeries under general anesthesia. Two patients did not meet the inclusion criteria, and three patients declined to participate (Figure 3). After giving an informed consent, 87 patients were enrolled and randomized. Three patients (all from the QLB group) experienced intraoperative or immediate postoperative complications involving massive bleeding that required conversion to open surgery (laparotomy) for hemostasis; these patients were excluded from the final analysis as per protocol. There were 45 patients in the ESP group (*n* = 45) and 39 in the QLB group (*n* = 39). A total of 48 patients underwent NSS and 36 underwent nephrectomies. In the QLB group, 23 patients had NSS and 16 had nephrectomies. The two groups were similar regarding other demographic and anesthesia-related characteristics (Table 1). Although a statistically significant difference was observed in ASA class distribution between the groups (*p*=0.021), this comparison was one of four baseline variables tested. After applying the Bonferroni correction for multiple comparisons (adjusted significance threshold *α* = 0.0125), this difference was no longer statistically significant.

The mean dosage of oxycodone, number of requested boluses, mean time of surgery, estimated blood loss, and mean dosage of remifentanil are presented in Table 2.

The total oxycodone consumption within the first 24 h after surgery (primary endpoint) did not differ significantly between the ESP and QLB groups. The median (IQR) 24 h oxycodone dose was 22.66 mg [10–30] in the ESP group versus 22.66 mg [12–30] in the QLB group (*p*=0.77). The mean number of patient-administered oxycodone boluses was also similar between groups (ESP: 17.47 ± 17.58 vs. QLB: 19.05 ± 17.91 bolus requests, *p*=0.84). There was no significant difference in intraoperative opioid requirements: the mean remifentanil usage during surgery was 823.24 ± 390.2 μg in ESP patients vs 856.64 ± 407.46 μg in QLB patients (*p*=0.427). Estimated blood loss and fluid administration volumes were comparable as well.

Pain was evaluated using the NRS at 1, 2, 6, 12, and 24 h after surgery. Postoperative pain intensity (NRS scores) at the predefined time points is presented in Table 3. Pain scores were well controlled in both groups throughout the 24 h period. There were no statistically significant differences in NRS at 1, 2, 6, 12, or 24 h between the ESP and QLB groups (not statistically significant). The results of the NRS evaluation are presented in Table 3.

PONV was present in seven patients in the ESP group and five patients in the QLB group. There was no statistically significant difference between the groups (*p*=0.964).

We considered the emergence time from anesthesia longer than 10 min from the end of the procedure to indicate prolonged awakening. One patient in the ESP group experienced prolonged awakening, while none of patients in the QLB group experienced prolonged emergence; however, this difference was not statistically significant (*p*=1).

No complications, such as hypotension, arrhythmia, or allergic reaction, were observed during the intra- or postoperative periods in any patient. Neither block-related complications nor side effects were observed postoperatively.

## 4. Discussion

The current study was designed to evaluate the perioperative analgesic efficacy of a preoperative ultrasound-guided single-injection QLB1 or ESP block in patients undergoing laparoscopic renal surgery under general anesthesia. In our clinic, regional techniques such as neuroaxial and plane blocks are preferred, especially in major abdominal surgeries. We did not encounter complications such as perforation, hematoma, infection, or significant hemodynamic instability. The main findings of this study indicate that both the ESP block and QLB1 provided similar and adequate postoperative analgesia in the early postoperative stage. There was no significant difference in opioid consumption between the two groups during the postoperative period. Additionally, we found no differences in NRS scores regardless of the type of block used. Similarly, intraoperative remifentanil consumption did not significantly differ between the groups.

Both groups in our study had relatively low pain scores and opioid requirements postoperatively, indicating adequate analgesia. The lack of a difference in outcomes between ESP and QLB1 may be explained by the mechanism of action and spread of LA in these blocks. The ESP block is known to cause extensive craniocaudal spread in the paraspinal plane, often reaching the thoracic paravertebral space and covering multiple dermatomes of both dorsal and ventral rami.

Similarly, the QLB1, though administered more laterally, can result in LA tracking medially along the thoracolumbar interfascial plane toward the paravertebral space. Thus, both blocks likely anesthetize the relevant neural pathways that innervate the laparoscopic nephrectomy incision sites (T6-L1 dermatomes) and possibly provide some visceral analgesia via spread to the sympathetic fibers. This comparable extent of LA spread and nerve coverage could account for the equivalent analgesic efficacy observed. Furthermore, we performed both blocks bilaterally, ensuring that any potential contribution of contralateral innervation or referred pain was mitigated in both groups. The bilateral application (while perhaps not strictly necessary for unilateral surgery) was used to maximize pain control and parity between techniques; as a result, patients in both groups received a very comprehensive regional block, potentially minimizing any difference between the two methods.

Zhang et al. [14] compared the analgesic effects of ESP block versus QLB following laparoscopic nephrectomies. They included 110 patients in both groups (QLB and ESP). The main finding was that the ESP block decreased 24 h sufentanil consumption by about 30% compared to the posterior approach QLB. The main difference between their methodology and ours in methodology was the type of QLB; we used the QLB1 approach, while Zhang et al. [14] used QLB2. Additionally, they administered sufentanil in the postoperative period instead of oxycodone. The sample sizes were similar, and the different results may be attributed to the different types of QLB.

However, the QLB can be used as an add-on block to reduce the requirement for general anesthesia medications intraoperatively or as a main component of multimodal analgesia postoperatively. The needle tip is placed at the anterolateral border of the QLB muscle at its junction with the transversalis fascia, and the LA is injected. Therefore, the QLB may be used to provide postoperative analgesia for the abdominal and pelvic regions. Evidence suggests possible improvement in visceral pain coverage [17]. Additionally, the location of the injection sites provides an element of safety, as the targets for injection are relatively distant from the peritoneal cavity, abdominal organs, and large blood vessels. All four QLB approaches have been used in patients undergoing laparoscopic nephrectomies and have been found effective when compared with no block or placebo [18–22].

In our study, we compared two blocks: ESP and QLB, and we found no difference in pain levels or opioid consumption between these groups of patients. The first study to compare QLB and the ESP blocks was performed by Aygun et al. [13], which included 80 patients after cholecystectomy. Their results showed no statistically significant difference in morphine consumption at the 1^st^, 6^th^, 12^th^, and 18^th^ hours postoperation. Although NRS scores were lower in the ESP group at the 1^st^ hour (*p* < 0.001), they were similar at the 6^th^, 12^th^, 18^th^, and 24^th^ hours after the operation. In our study, we found no statistically significant difference in NRS scores after surgery. The main difference between our study and that of Aygun et al. was the localization of the drug injected for QLB. We preferred QLB1, where the LA was deposited on the anterolateral QL muscle (between the deep and middle layers of the thoracolumbar fascia). Aygun et al. utilized the QLB2 modification, where the LA was given on the posterolateral QL muscle (between the superficial and middle layers of the thoracolumbar fascia).

Baran [23] compared QLB and the ESP blocks in women after hysterectomy, including 91 patients divided into three groups: the ESP block, the anterior QLB, and control group. In the ESP and QLB groups, visual analog scale (VAS) scores at 2, 6, and 12 h postoperatively were significantly lower than those in the control group. However, VAS scores at these time points postoperatively did not differ significantly between the ESP and QLB groups. The opioid consumption in the QLB and ESP groups was also significantly lower than in the control group. The incidence of postoperative nausea and vomiting was significantly lower in the QLB and ESP groups compared to that of the control group; however, it did not differ significantly between the ESP and QLB groups. In our study, we did not find significant differences in occurrence rates of postoperative nausea and vomiting between the studied groups.

A comparison of two blocks was studied by Kamel et al. [24]. They included 48 women after hysterectomies: half received the ESP block, and other 24 women received the TAP block. VAS scores were significantly lower in the ESP group compared to the TAP group, with statistically lower scores at 30 min, 2, 12, 16, 20, and 24 h postoperatively (*p* < 0.0001). While the TAP block is an easy technique that decreases postoperative pain and opioid consumption, it lacks visceral pain relief and limits the spread of LAs [25].

Onay et al. [15] compared QLB2 with the ESP block in patients after open nephrectomies. They included 40 patients and found results similar to ours regarding pain scores and morphine consumption. They found that the median pain scores were 1–2 points lower (on an 11-point NRS) in patients given the ESP block within 24 h, but differences were not statistically significant due to the small sample size. It is worth noting that we included twice as many patients with similar findings.

The ESP block exerts its effects by spreading LAs to the thoracic paravertebral space, epidural space, and dorsal ramus [12]. Under the guidance of ultrasonography, the ESP block can be performed easily and safely, even in obese patients [26]. The meta-analysis including 25 randomized controlled trials reported that the use of the ESP block reduced the postoperative pain scores at rest or with activity compared to the control group (no block or sham block) [27]. In the study of Fan et al. [28], it was demonstrated that the ESP block provides noninferior analgesia for pain at rest within 24 postoperative hours in comparison to the thoracic paravertebral block for laparoscopic nephrectomy. The number of patients in their study (60 patients) was lower than in ours, but the results were similar. They also did not detect differences in the PONV scores between groups.

There was no difference in the secondary endpoints between groups.

## 5. Limitations

The main limitation was the lack of a control group. We designed this study as a simple randomized controlled trial. Given that many studies showed that blocks reduce opioid consumption and NRS scores, we decided to omit the control group. Anesthesiologists were not masked to the trial intervention. However, the investigators responsible for postoperative assessment as well as patients and other healthcare providers were not aware of group assignments. Additionally, although none of the patients presented with LA systemic toxicity, further studies are needed to validate optimal LA concentrations and volumes. Finally, although all blocks were performed by a single experienced anesthesiologist, there is always some degree of variability in technique (e.g., slight differences in needle angle or exact injectate plane) that could affect outcomes. We did not track ultrasound images or the precise extent of spread in each case, so we cannot analyze the relationship between block anatomy and analgesia in detail. Intraoperatively, anesthetic management (remifentanil infusion, etc.) was standardized by a protocol, but small variations in surgical stress or anesthetic depth could influence immediate postoperative pain for some patients. We attempted to mitigate this by randomization and found no baseline differences in surgical factors (like operative time or blood loss) between groups.

One potential limitation noted was the uneven distribution of ASA physical status classes between the groups. However, this difference lost statistical significance after the Bonferroni correction for multiple comparisons, and all patients were classified as ASA I–III, with no severe systemic illness present. Therefore, the groups can still be considered clinically comparable, and we believe this does not substantially affect the internal validity or generalizability of our findings.

Future directions: Further research could explore whether a unilateral block (on the surgical side only) might suffice for laparoscopic nephrectomy, as this would halve the LA dose and procedure time. One reason we chose bilateral blocks was to ensure any referred pain (e.g., shoulder pain from diaphragmatic irritation or pain from ancillary port sites) was also addressed; however, unilateral approaches could be tested in the future to see if analgesia remains adequate. Additionally, comparing different approaches of QLB (QLB1 vs. QLB2) head-to-head in similar surgeries would be valuable to determine if one variant has an advantage. As suggested by recent data, the posterior QLB2 may not spread as consistently to cover lower thoracic segments as an ESP or a QLB1 does, which could explain differences in outcomes. Thus, identifying the optimal QLB approach for a given surgery is important. Finally, studies with longer follow-up could assess if there are any differences in block duration or late outcomes (48 h or beyond), as our study focused on the first 24 h post-op.

## 6. Conclusion

The effects of ultrasound-guided bilateral QLB1 and ESP blocks in patients undergoing either partial or radical laparoscopic nephrectomies were found to be similar in terms of postoperative pain and opioid consumption. Given their comparable efficacy and safety, the choice between ESP and QLB for laparoscopic nephrectomy can be based on practitioner expertise and logistical considerations. These findings support the inclusion of either ESP or QLB1 block as part of a multimodal analgesia regimen to enhance recovery after minimally invasive kidney surgery. Further studies are warranted to confirm these results in larger populations to compare different QLB approaches and to refine patient selection for each technique. Our results add to the growing evidence that ESP and QLB blocks are valuable tools in the management of postoperative pain for abdominal surgeries, helping to reduce reliance on opioids and improve patient comfort.

## Figures and Tables

**Table 1:** Demographic and anesthesia-related characteristics.

Parameter		ESP (*N* = 45)	QLB (*N* = 39)	Total (*N* = 84)	*p*
BMI (kg/m^2^)	Mean (SD)	28.05 (5.44)	27.58 (4.43)	27.84 (4.97)	0.436

Age (years)	Mean (SD)	56.8 (14.95)	59.33 (13.42)	56.9 (14.35)	0.75

ASA	ASA I	3 (6.67%)	0 (0%)	3 (3.57%)	0.021^∗^
	ASA II	32 (71.11%)	24 (61.54%)	56 (66.67%)	
	ASA III	10 (22.22%)	15 (38.46%)	25 (29.76%)	

Type of surgery	NSS	25 (55.56%)	23 (58.97%)	48 (57.14%)	0.378
	Nephrectomy	20 (44.44%)	16 (41.03%)	36 (42.86%)	

^∗^After the Bonferroni correction (adjusted *α* = 0.0125), ASA class does not meet the threshold for statistical significance.

**Table 2:** Number of requested boluses, mean time of surgery, value of intraoperative bleeding, and mean dosage of remifentanil.

Parameter		ESP (*N* = 45)	QLB (*N* = 39)	*p*
Mean dosage of remifentanil (μg)	Mean (SD)	823.24 (390.2)	856.64 (407.46)	*p*=0.427
	Median (quartiles)	700 (500–1000)	780 (617.5–1000)	
	Range	350–2100	387–2750	
	*n*	45	39	

Number of given boluses of oxycodone	Mean (SD)	11.33 (7.35)	11.33 (7.87)	*p*=0.77
	Median (quartiles)	12 (5–15)	10 (6–15)	
	Range	0–36	0–36	
	*n*	45	39	

Number of requested boluses of oxycodone	Mean (SD)	17.47 (17.58)	19.05 (17.91)	*p*=0.84
	Median (quartiles)	14 (5–22)	12 (6–26.5)	
	Range	0–100	0–64	
	*n*	45	39	

Time of surgery (minutes)	Mean (SD)	116.33 (37.89)	130.9 (48.37)	*p*=0.186
	Median (quartiles)	120 (85–130)	120 (102.5–150)	
	Range	55–205	45–300	
	*n*	45	39	

Estimated blood loss (ml)	Mean (SD)	213.33 (241.3)	212.82 (188.04)	*p*=0.502
	Median (quartiles)	150 (100–300)	200 (100–300)	
	Range	0–1200	0–1000	
	*n*	45	39	

**Table 3:** NRS scores at 1, 2, 6, 12, and 24 h after surgery and a total score.

Parameter		ESP (*N* = 45)	QLB (*N* = 39)	*p*
NRS w 1 h	Mean (SD)	3.36 (2.81)	3.03 (2.92)	*p*=0.567
	Median (quartiles)	3 (1–5)	2 (0.5–5)	
	Range	0–10	0–10	
	*n*	45	39	

NRS w 2 h	Mean (SD)	3 (2.08)	2.9 (2.12)	*p*=0.693
	Median (quartiles)	3 (2–4)	3 (1.5–4)	
	Range	0–8	0–8	
	*n*	45	39	

NRS w 6 h	Mean (SD)	2.73 (2.28)	2.41 (1.76)	*p*=0.757
	Median (quartiles)	2 (1–4)	2 (1–4)	
	Range	0–8	0–5	
	*n*	45	39	

NRS w 12 h	Mean (SD)	2.49 (2.6)	2.18 (2.01)	*p*=0.887
	Median (quartiles)	2 (0–4)	2 (0.5–3.5)	
	Range	0–10	0–7	
	*n*	45	39	

NRS w 24 h	Mean (SD)	2.14 (1.97)	1.82 (1.75)	*p*=0.506
	Median (quartiles)	2 (0.75–3)	1 (0–3)	
	Range	0–8	0–7	
	*n*	45	39	

Total NRS	Mean (SD)	13.67 (8.44)	12.33 (7.59)	*p*=0.404
	Median (quartiles)	12 (8–17)	9 (6–18)	
	Range	0–33	0–29	
	*n*	45	39	

## Data Availability

The datasets analyzed during the current study are available from the corresponding author on reasonable request.
